# Transcriptomic and physiological analyses of *Medicago sativa* L. roots in response to lead stress

**DOI:** 10.1371/journal.pone.0175307

**Published:** 2017-04-07

**Authors:** Bo Xu, Yingzhe Wang, Shichao Zhang, Qiang Guo, Yan Jin, Jingjing Chen, Yunhang Gao, Hongxia Ma

**Affiliations:** 1 College of Animal Science and Technology, Jilin Agricultural University, Changchun, Jilin, China; 2 Agro-Biotechnology Research Institute, Jilin Academy of Agricultural Sciences, Gongzhuling, Jilin, China; Nanjing Agricultural University, CHINA

## Abstract

Lead (Pb) is one of the nonessential and toxic metals that threaten the environment and human health. *Medicago sativa* L. is a legume with high salt tolerance and high biomass production. It is not only a globally important forage crop but is also an ideal plant for phytoremediation. However, the biological and molecular mechanisms that respond to heavy metals are still not well defined in *M*. *sativa*. In this study, *de novo* and strand-specific RNA-sequencing was performed to identify genes involved in the Pb stress response in *M*. *sativa* roots. A total of 415,350 unigenes were obtained from the assembled cDNA libraries, among which 5,416 were identified as significantly differentially expressed genes (DEGs) (false discovery rate < 0.005) between cDNA libraries from control and Pb-treated plants. Gene ontology and Kyoto Encyclopedia of Genes and Genomes (KEGG) pathway enrichment analyses of the DEGs showed they mainly clustered with terms associated with binding, transport, membranes, and the pathways related to signal and energy metabolism. Moreover, a number of candidate genes included antioxidant enzymes, metal transporters, and transcription factors involved in heavy metal response were upregulated under Pb stress. Quantitative real-time PCR(qRT-PCR) validation of the expression patterns of 10 randomly selected candidate DEGs were consistent with the transcriptome analysis results. Thus, this study offers new information towards the investigation of biological changes and molecular mechanisms related to Pb stress response in plants.

## Introduction

Metal contaminants such as lead (Pb), cadmium (Cd), and mercury (Hg) are nonessential elements released into the environment by anthropogenic activities and are toxic to plants and can threaten human health through the food chain [1, 2]. Pb is a highly toxic heavy metal that can be absorbed by plants; inhibit root and shoot growth; cause leaf chlorosis; and disturb other physiological processes such as mitosis, transpiration, and DNA synthesis [3, 4]. Physiological disorders and ultimately death can result from the production of large quantities of reactive oxygen species (ROS), which are induced by heavy metals and can cause damage to proteins and DNA in plant cells [2, 5].

Techniques such as soil washing, soil leaching, and phytoremediation have been used to remediate soil contaminated with heavy metals [6], of which phytoremediation is generally accepted as an efficient, cost-effective, and environmentally friendly approach to clean up metal-contaminated soil [6–9]. Thus, understanding the gene expression and regulation that are components of the response to heavy metal toxicity in plants is important for understanding the genetic and molecular mechanisms involved in phytoremediation.

Previous studies have identified a group of gene families and associated proteins that are involved in metal transport, including iron-responsive transport proteins (IRT) [10–12], cation diffusion facilitators (CDFs) [13], P-type ATPases [14], ATP-binding cassette (ABC) transporters, natural resistance-associated macrophage proteins (NRAMP) [15, 16], and the F-box family [17]. The absorption and accumulation of heavy metals can also trigger a stress response in plants; for example, it can stimulate the expression of transcription factors (TFs) such as myeloblastosis proteins (MYB) and ethylene-responsive factors (ERFs) and activate signaling proteins such as mitogen-activated protein kinases (MAPKs) and calcium-binding proteins [18, 19]. In addition, the activities of antioxidant enzymes and the metabolism of antioxidants such as glutathione and flavonoids significantly increase in plants exposed to heavy metal stress [4, 19, 20]. Genes involved in heavy metal responses have been widely studied in different plant species [15, 21–24]; however, the genome-wide network for gene expression and interactions in response to heavy metals are still relatively unknown. Recent studies using next-generation sequencing technology identified a number of microRNAs and their corresponding targets, which were genes whose expression is sensitive to heavy metal stress [20, 25–27]. These findings indicate that the response induced by heavy metals in plants is a complex network that involves diverse physiological processes and metabolic pathways.

*Medicago sativa* L. is a salt-tolerant plant with a highly branched root system, high biomass production, and wide adaptability to multiple environments. These characteristics make *M*. *sativa* an ideal soil amendment plant. Understanding the molecular basis for *M*. *sativa* responses to heavy metals will facilitate its application in phytoremediation. In this study, the activities of antioxidant enzymes superoxide dismutase (SOD), peroxidase (POD), and catalase (CAT) were determined in (Pb(NO_3_)_2_)*-*treated *M*. *sativa* roots and compared with those of the untreated control plants. Transcriptional profiling of roots from Pb-treated and control *M*. *sativa* plants highlighted the quantity of genes that are involved in the Pb stress response.

## Results

### Phenotypic and physiological responses to Pb in *M*. *sativa*

**Fig 1:**
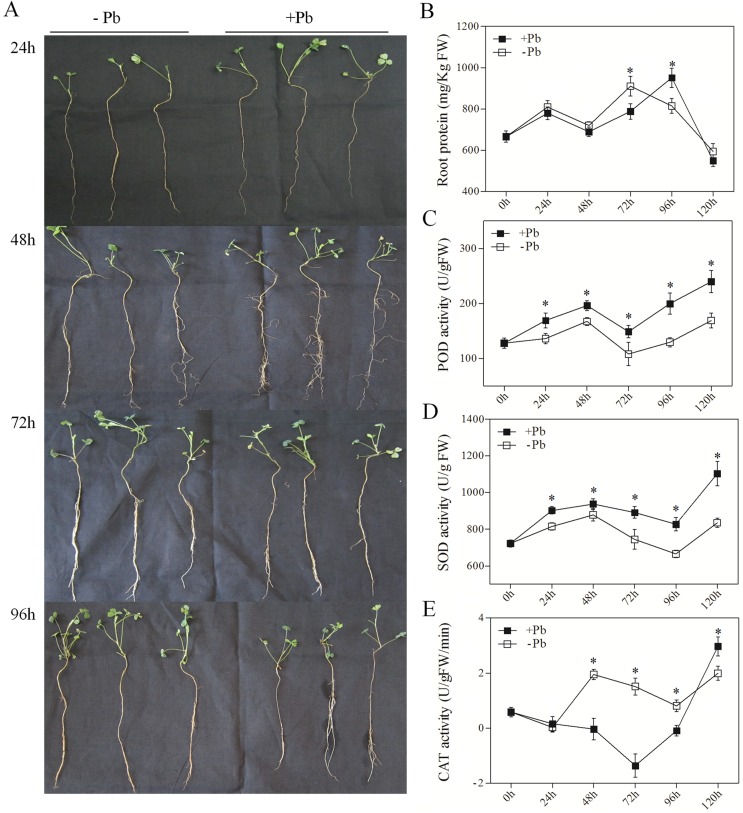
Phenotypic and physiological responses of *M*. *sativa* to Pb. (A) The phenotypic response of control and Pb-treated *M*. *sativa* at 24 h, 48 h, 72 h, and 96 h, respectively. (B) Measurement of the protein content in roots of control and Pb-treated plants. (C–E) The enzyme activities of peroxidase (POD), superoxide dismutase (SOD), and catalase (CAT) in control and Pb-treated *M*. *sativa* at 0 h, 24 h, 48 h, 72 h, and 96 h, respectively. Stars indicate significant differences (α = 0.05).

The effect of Pb (200 mg/L Pb(NO_3_)_2_) on *M*. *sativa* after treatment for 24 h, 48 h, 72 h, and 96 h are presented in Fig 1. The seedlings showed obvious growth inhibition both in the roots and shoot after 96 h, the protein content in seedlings significantly decreased after 48 h, and the activities SOD, POD, and CAT increased after 48 h until reaching a peak after 72 h (Fig 1). These results suggest that Pb affects both growth and biological activities in *M*. *sativa* over time.

### Transcriptome assembly and functional annotation

A total of 42 Gb of data, equivalent to 282,999,744 clean reads were generated from the five cDNA libraries (Table 1). The GC content of the five libraries ranged from 42.46% to 43.08%, and the Q20 percentages ranged from 97.99% to 98.27%. The mapped reads for each library ranged from 58.13% to 62.82%. The generated unigenes for each library ranged from 232,083 to 265,110.

**Table 1:** Throughput and quality of strand-specific RNA-seq of the five *Medicago sativa* libraries.

Library ID	Clean Reads	Clean Base	Clean GC%	Clean Q20%	Total mapped reads (%)	Unigene
**C0**	52,761,700	7.83G	42.46	98.15	32,362,486(61.33)	252,268
**C48**	54,525,692	8.09G	42.76	97.99	31,693,092(58.13)	243,292
**C96**	60,510,310	8.99G	42.49	98.25	36,841,382(60.88)	255,256
**Pb48**	62,584,610	9.31G	43.08	98.27	39,316,818(62.82)	265,110
**Pb96**	52,617,432	7.82G	42.83	98.13	31,888,382(60.6)	232,083

The *de novo* assembly of all the clean reads generated 508,781 contigs with an average length of 473.95 bp and N50 length of 519 bp (Table 2). The minimum and maximum lengths were 201 bp and 14,904 bp, respectively. In total, 415,350 unigenes were obtained with an average length of 426.68 bp and N50 length of 436 bp. RNA-seq data quality analysis showed more than 80% of unigenes had coverage above 50% for each library (S1 Fig).

**Table 2:** Summary of assembled RNA-seq data.

Summary	Contig	Unigene
Total number	508,787	415,350
Average length (bp)	473.95	426.68
Min length (bp)	201	201
Max length (bp)	14,904	14,904
N50 length (bp)	519	436

Functional annotation of unigenes is shown in S2 Fig. In total, 113,139 (27.24%), 155,233 (37.37%), 271,695 (65.41%) and 101,719 (24.49%) unigenes (E-value < le^-5^) were significantly matched to the COG, NR Swiss-Prot, and KEGG database, respectively (S2 Fig). Most of the unigenes (71.86%) matched *Medicago truncatula* in the NR database, suggesting the sequences of the *M*. *sativa* transcripts obtained in this study were correctly assembled and annotated. The COG-matched unigenes were clustered into 26 functional categories and the top three categories were “general function prediction only”; “signal transduction mechanism”; and “posttranslational modification, protein turnover, and chaperones” (S3 Fig).

### Identification of differentially expressed genes (DEGs)

**Fig 2:**
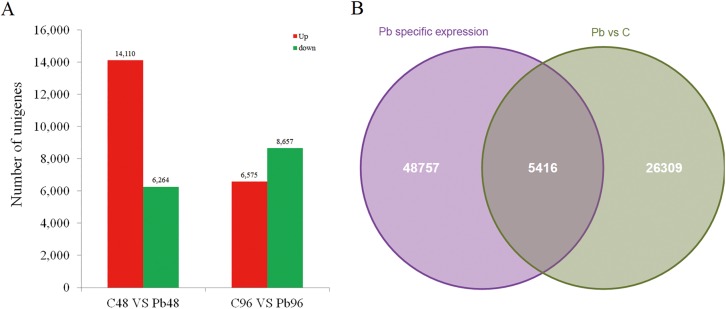
Pairwise comparison of unigene expression between cDNA libraries from the control and the Pb-treated plants (A). A Venn diagram of the differentially expressed genes (DEGs) specific to Pb-treated roots (B).

To identify unigenes induced by Pb stress, the five libraries were first divided into a control and a treatment group, C0-C48-C96 and C0-Pb48-Pb96, and a comparison of the two groups identified 54,173 unigenes that were specifically expressed in the C0-Pb48-Pb96 group. Next, a pairwise comparison was performed between libraries C48 and Pb48, and C96 and Pb96, to identify DEGs at specific times after Pb exposure. A total of 31,725 DEGs were identified between Pb-treated and control libraries (Fig 2A). Finally, a comparison between the 54,173 unigenes specifically expressed in the Pb-treated library and the 31,725 DEGs identified 5,416 upregulated DEGs under Pb stress (Fig 2B, S2 Table). Among these 5,416 DEGs, 3,609 (66.64%) were annotated; of these DEGs, 1,749 unigenes were mapped to the gene ontology (GO) database and 792 of these unigenes mapped to 196 KEGG pathways (S2 Table). Of the 5,416 DEGs specific to Pb treatment, 3,215 were specifically expressed in Pb48, 1,265 were specifically expressed in Pb96, and the last 936 were expressed in both Pb48 and Pb96 libraries (S2 Table). The large number of DEGs indicates that Pb treatment alters gene expression across the *Medicago* genome, which may result in specific stress responses to Pb exposure. In addition, the differences of DEGs specifically expressed in Pb48 or Pb96 suggest extensive biological changes in *Medicago* after Pb treatment.

### GO and KEGG enrichment analysis of DEGs

**Fig 3:**
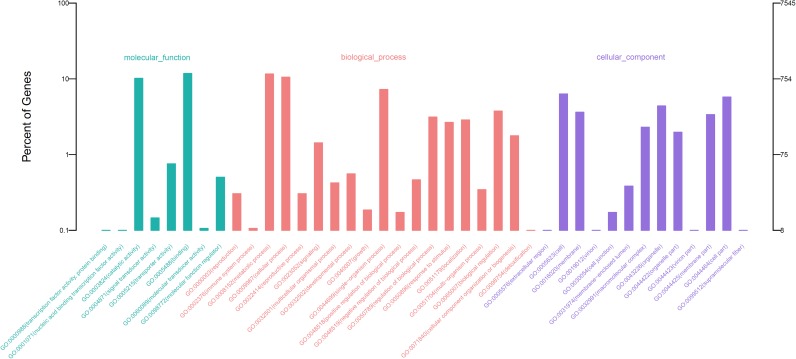
Gene ontology classification of DEGs. The *x-axis* represents GO terms belonging to three categories, the left *y-axis* represents gene percentages of each term, and the right *y-axis* represents gene numbers of each term.

GO annotation classified 1,749 Pb-induced DEGs into 39 level 2 GO terms (Fig 3). These GO terms included binding (GO:0005488) and catalytic activity (GO:0003824) in the molecular function category, metabolic process (GO:0008152), cellular process (GO:0009987), and the single-organism process (GO:0044699) in the biological process category, and the cell (GO:0005263) and cell part (GO:0044464) in the cellular component category. Further GO enrichment identified 83 molecular function terms, 229 biological process terms, and 42 cellular component terms that were significantly enriched in the 1,749 Pb-induced DEGs (*P* < 0.05) (S3 Table). The most frequently represented terms in the molecular function category were binding (897, 51.29%, GO:0005488), nucleotide binding (410, 23.44%, GO:0000166), and carbohydrate derivative binding (365, 20.87%, GO:0097367). For the biological process category, the most represented terms were cellular macromolecule metabolic process (445, 25.44%, GO:0044260), localization (216, 12.35%, GO:0051179), establishment of localization (214, 12.24%, GO:0051234), and transport (210, 12.01%, GO:0006810). For the cellular component category, the most abundant terms were membrane (344, 19.67%, GO:0016020) and intrinsic component of membrane (243, 13.89%, GO:0031224). The classification of these unigenes indicated that binding activity, metabolic activity, transport activity, and membrane function relate to the Pb stress response in *M*. *sativa*.

KEGG enrichment analysis classified 792 Pb-induced DEGs into 247 pathways, 10 of which were significantly enriched at *P* < 0.05 (S4 Table). The enriched pathways are shown in Table 3, including signaling pathways such as “PPAR signaling pathway” (ko03320; 28, 3.54%) and “plant hormone signal transduction” (ko04075, 14, 1.77%); physiological and biochemical processes for “meiosis-yeast” (ko04113; 21, 2.65%), “pentose and glucuronate interconversions” (ko00040, 27, 3.41%), “citrate cycle (TCA cycle)” (ko00020, 20, 2.53%), “photosynthesis-antenna proteins” (ko00196, 10, 1.26%), and “glycolysis/gluconeogenesis” (ko00010, 32, 4.04%); and secondary metabolic pathways for “caffeine metabolism” (ko00232, 5, 0.63%), “anthocyanin biosynthesis” (ko00942, 1, 0.13%), and “cyanoamino acid metabolism” (ko00460, 4, 0.51%). These indicate that in *M*. *sativa*, Pb stress mainly influences pathways related to signaling, cell growth, energy metabolism, and secondary metabolism.

**Table 3:** KEGG pathways of significantly enriched for differentially expressed genes.

Pathway	DEG number (792)	*P*-value	Pathway ID
PPAR signaling pathway	28(3.54%)	1.13E-05	ko03320
Meiosis—yeast	21(2.65%)	0.000254079	ko04113
Pentose and glucuronate interconversions	27(3.41%)	0.001000734	ko00040
Caffeine metabolism	5(0.63%)	0.001189044	ko00232
Glycolysis / Gluconeogenesis	32(4.04%)	0.00380755	ko00010
Citrate cycle (TCA cycle)	20(2.53%)	0.011745552	ko00020
Anthocyanin biosynthesis	1(0.13%)	0.011973875	ko00942
Cyanoamino acid metabolism	4(0.51%)	0.02386785	ko00460
Photosynthesis—antenna proteins	10(1.26%)	0.027637894	ko00196
Plant hormone signal transduction	14(1.77%)	0.044580993	ko04075

### Candidate genes related to Pb stress

Pb stress leads to an increased level of ROS in plants, and thus the activities of antioxidative enzymes such as CAT, POD, and SOD increase in plants exposed to Pb stress [28, 29]. Here, eleven DEGs coding for POD and four DEGs coding for SOD were predicted to be involved in Pb stress (S5 Table). In addition to the antioxidant enzymes, the biosynthesis of antioxidants was also elevated in response to Pb stress in *M*. *sativa*. Glutathione is one of the most important plant antioxidants and is also a substrate for phytochelatins, which are metal-chelating compounds that enable metal tolerance [4, 27]. We identified 15 DEGs coding for glutathione S-transferase (GST) in the “glutathione metabolism” (ko00480) pathway (S5 Table). Production of secondary metabolites like flavonoids and isoflavonoids is an effective mechanism adopted by plants against heavy metal toxicity [4]. In this study, 14 DEGs involved in flavonoid and isoflavonoid synthesis, including three chalcone synthase (CHS) genes that are involved in the first step of flavonoid biosynthesis, were identified in the flavonoid-related pathways (ko00940, ko00941, ko00942, ko00943, and ko00944) (S5 Table).

Metal transporters and stress response proteins play vital roles in heavy metal tolerance in plants [19]. In this study, we identified a number of DEGs coding for metal transporters, including 16 DEGs for ABC transporters, three DEGs for IRT, three DEGs for CDFs, and two DEGs for heavy metal transport (S5 Table). Signal transduction and expression of TFs are also responsive to stress in plants. We identified 13 DEGs for calcium-binding proteins or MAPKs. Additionally, 24 DEGs for TFs in the WRKY family, MYB ethylene-responsive transcription factor (ERF), and bZIP were identified in Pb-treated *M*. *sativa* (S5 Table).

**Fig 4:**
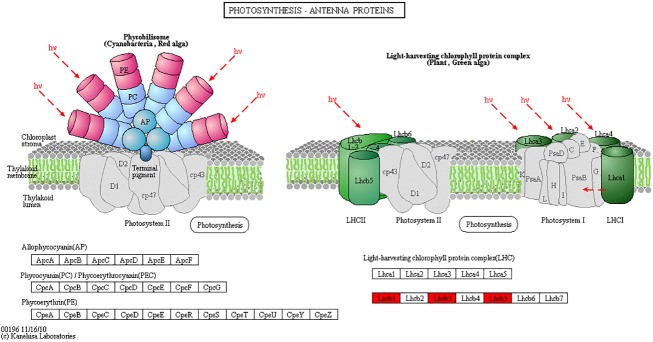
Differentially expressed genes involved in “photosynthesis-antenna proteins.” The red column indicates the upregulated enzymes under Pb stress. This color-coded map of DEGs corresponds to map00196 in the KEGG database (http://www.genome.jp/dbget-bin/www_bget?pathway:map00196).

Previous studies have shown that photosynthesis is sensitive to heavy metal stress [27]. In this study, the KEGG pathways for “photosynthesis—antenna proteins” (ko00196) were significantly enriched in Pb-stress cDNA libraries (Table 3), in which the expression levels of 10 DEGs coding for chlorophyll a/b-binding protein (light-harvesting complex I protein) were significantly upregulated (S5 Table). The DEGs that were upregulated in the “photosynthesis—antenna proteins” pathway are shown in Fig 4.

### qRT-PCR validation of candidate DEGs

**Fig 5:**
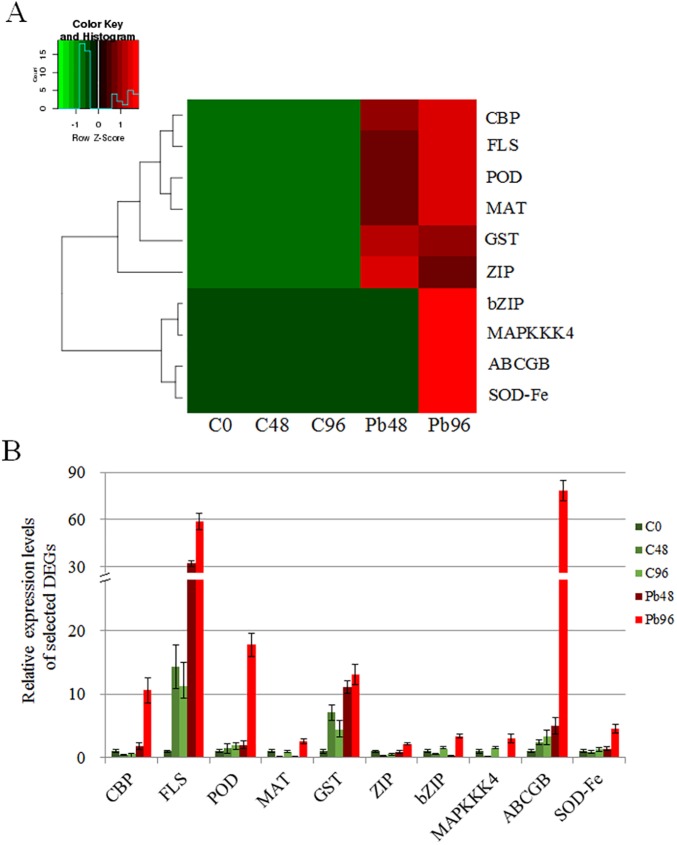
Heat map of expression levels of 10 DEGs by FPKM (A) and expression levels by qRT-PCR analysis (B).

To evaluate the quality of the transcriptome data generated by the Illumina high-throughput sequencing platform, ten candidate DEGs that were predicted to be involved in the Pb stress response were selected for validation using qRT-PCR analysis (S1 Table). For these genes in control and Pb-treated plants, the expression levels calculated by qRT-PCR were consistent with the expression levels from the transcriptome sequencing data (Fig 5). This indicates that our transcriptome sequencing data are reliable.

## Discussion

Pb is now a widespread soil contaminant all over the world and poses a great threat to food safety and human health [30, 31]. Different physical, chemical, and biological methods have been employed to address heavy metal pollution in the environment, among which phytoremediation is thought to be an efficient, cost-effective, and environmentally friendly method [32, 33]. However, the genes involved in protection against heavy metal toxicity and the molecular mechanisms underlying this protection are still undefined in plants. In this study, we identified a number of genes and pathways related to Pb stress responses in *M*. *sativa* using transcriptome sequencing, which improves our current knowledge of heavy metal responses and tolerance in plants.

*De novo* transcriptome sequencing and assembly had been widely used to evaluate gene expression levels, discover novel genes and alternative splicing in non-model plants. Here, *de novo* transcriptome sequencing of *M*. *sativa* roots generated 415,350 unigenes with an average length of 426.7 bp, representing 177,221,538 bp (177.2 Mb) of the sequence. The N50 of unigene was 436 bp, this is lower than those of other *M*. *sativa* transcriptome sequencing projects of Zhang *et al*. (1,451 bp) [34], and Gao *et al*. (1,117 bp) [35], however, close to those of Yang *et al*. (289 bp) [36], and Postnikova *et al*. (591 bp) [37]. N50 value is an indicator of the transcript contiguity of *de novo* assembly. It is affected by several factors, such as species, assembly software, and assembly parameters, especially *k*-mer value [36, 37]. Thus, the different N50 values among studies may result from different software and parameters. Although the mean length and N50 value of our study was relatively lower than many other studies, this did not affect the accuracy of the transcriptome, as 71.86% of the unigenes match to the genome of *M*. *truncatula*, the closely related species of *M*. *sativa* and the model leguminous plant.

The transcript levels of genes are used to reflect the biological differences between tissues and plant developmental stages. DEG identification and the subsequent GO and KEGG enrichment analyses of the cDNA libraries from control and Pb-treated plants can be directly related to the global biological changes and molecular mechanisms that respond to Pb in *M*. *sativa* roots. In this study, we identified 5,416 DEGs that were specifically expressed in the Pb-treated roots. The GO term and KEGG enrichment analyses showed that terms for transport (GO:0006810) and membrane (GO:0016020, GO:0031224) (S3 Table), and pathways related to signal (ko03320, ko04075) and energy metabolism (ko00040, ko00020, ko00196, ko00010) (Table 3), were significantly enriched in the identified DEGs. These results were in agreement with previous studies that reported that heavy metals damaged plant membrane systems, inhibited photosynthesis, and delayed plant growth [27, 38–40].

The plasma membrane is the primary environmental barrier for a plant cell and mediates the exchange of information and materials between the cell interior and the extracellular environment [41, 42]. Thus, the enzymes and transporters located on the plasma membrane play essential roles in heavy metal tolerance and detoxification. The membrane proteins in the natural resistance-associated macrophage proteins and ABC transporter families, and ATPases, are involved in metal tolerance by transporting metals to the vacuole or pumping them back out of the cells [39, 43–45]. Additionally, membrane-associated signaling proteins, such as MAPKs and calcium-binding related protein kinases, are involved in several stress responses and are activated under heavy metal stress [20, 46]. In this study, a number of DEGs coding membrane proteins associated with metal transport and signaling were identified, including 16 DEGs for metal transporters (ABC transporters, CDFs, and zinc-regulated transporter, IRT-like proteins), seven DEGs coding for chlorophyll a/b-binding proteins (light-harvesting complex), and 13 DEGs coding for signaling proteins (MAPKs and calcium-binding protein) (S5 Table). This indicates that the membrane and its associated transport proteins are important in Pb response and tolerance in *M*. *sativa*.

Photosynthesis is the most important biological process for plants, and heavy metals like Pb inhibit photosynthesis by decreasing the chlorophyll content and number of chloroplasts, and disrupting chloroplast structure [27, 47, 48]. The chloroplast chlorophyll a/b-binding protein is an essential factor in photosynthesis that participates in the two key processes of light absorption and electron transfer [27, 49]. In this study, the DEGs related to “photosynthesis—antenna proteins” (ko00196) in our pathway prediction analysis were significantly enriched. Among the 10 DEGs coding for the chlorophyll a/b-binding protein, nine were upregulated after 48 h treatment with Pb (S5 Table). However, they were downregulated after 96 h, which may indicate that Pb stress stimulates the expression of chlorophyll a/b-binding protein, while the excess accumulation of Pb in plant cells can lead to an inhibition of chlorophyll a/b-binding protein expression and affect photosynthesis.

Antioxidant synthesis is a main mechanism for protection against heavy metal toxicity in plants through their ROS-scavenging activity [2, 50]. Antioxidant enzymes such as SOD, POD, and CAT play essential roles in controlling the ROS level in cells [51]. Studies of heavy metal tolerance in different plant species show that the activities of SOD, POD, and CAT significantly increase after treatment with toxic metals [28, 29, 52, 53]. In this study, we demonstrated that the activities of SOD and POD significantly increased in the roots of Pb-treated *M*. *sativa* (Fig 1). In addition, the transcriptome data showed that 11 DEGs for POD and four DEGs for SOD were upregulated in Pb-treated roots (Fig 3, S5 Table). These results show that antioxidant enzymes act to reduce ROS damage in *M*. *sativa* after heavy metal exposure.

In addition to antioxidant enzymes, many kinds of metabolic products are efficient antioxidants in plant cells. Flavonoids and glutathione are well-documented antioxidants that protect against heavy metal toxicity [4, 19, 50, 54]. Flavonoids act as ROS scavengers and metal chelators, and isoflavonoids act as phytoalexins in response to heavy metals [4]. Glutathione plays an important role in heavy metal detoxification [19, 50] and is not only an important antioxidant but also participates in metal transport processes [4, 45, 55]. In this study, both transcriptome data and qRT-PCR analysis indicated that DEGs that encode flavonoid synthase genes were significantly upregulated under Pb stress (Fig 5, Table 3, S5 Table). Moreover, in accordance with previous reports, the DEGs for enzymes involved in the “glutathione metabolism” (ko00480) pathway were significantly upregulated in plants under Pb stress. Taken together, our results indicate that the antioxidant reactions are significantly activated to protect against Pb toxicity in *M*. *sativa* roots.

In summary, the *de novo* transcriptome sequencing of *M*. *sativa* roots obtained 415,350 unigenes, among which 5,416 were defined as DEGs that were specifically expressed in Pb-treated cDNA libraries. GO and KEGG pathway enrichment analyses of these DEGs showed that these genes mainly clustered with terms for binding, transport, and membrane, and the pathways were related to signaling and energy metabolism. Furthermore, a number of genes involved in heavy metal response were successfully identified. This study adds to the foundational knowledge of the biological changes and molecular mechanisms that respond to Pb stress in *M*. *sativa* roots. In addition to the previous studies on heavy metal response in different plant species, this will promote the use of phytoremediation of heavy metal-contaminated soils.

## Materials and methods

### Plant material, growth conditions, and treatments

Alfalfa seeds (*M*. *sativa* L. ‘Longdong’) were surface-sterilized, rinsed, and germinated in the dark at 26°C. Seedling were then grown in soil and supplied with 1/2 strength modified Hoagland nutrient solution [52]. Seedlings were grown under a 14 h light/10 h dark photoperiod with 70% humidity at 26°C. After 30 d, the seedlings were divided into two groups and treated with 0 or 200 mg/L Pb^2+^ as Pb(NO_3_)_2_ in 1/2 strength modified Hoagland nutrient solution for 0, 24, 48, 72, 96, or 120 h. The roots of seedlings were then harvested, surface cleaned, and frozen immediately in liquid nitrogen before being stored at -80°C for future analysis.

### Assay of soluble protein content and enzyme activity

The soluble protein content and antioxidant enzyme activities of SOD, POD, and CAT in roots of the Pb-treated and control groups were analyzed at each time point. Frozen root tissues were homogenized in 0.05 M phosphate buffer (pH = 7.8) and the homogenate was centrifuged at 15,000 ×*g* for 20 min at 4°C. The supernatant was used as a crude sample for enzymatic activity assays.

The soluble protein content was determined as descripted by Bradford [56], using Coomassie brilliant blue G-250 as a dye and albumin as a standard. The activity of SOD and CAT were assayed according to Li et al. [57]. One unit of SOD was defined as the enzyme amount that caused 50% inhibition reduction in nitro-blue tetrazolium. The enzyme activity of CAT was expressed as micrograms of H_2_O_2_ destroyed per minute per gram fresh weight (FW) root material. The activity of POD was assayed according to Fang and Kao [58]. A unit of POD specific activity was defined as enzyme units per gram FW root material.

### Library construction and sequencing

Total RNA from roots of Pb-treated and control plants at 0, 48, and 96 h (notated as Pb48, Pb96, C0, C48, and C96, respectively) were isolated using an RNAprep Pure Plant kit (BioTeke, China). The concentration and quality of RNA were assessed using Qubit 3.0 (Thermo Scientific, USA) and an Agilent 2100 Bioanalyzer (Agilent Technologies, USA), respectively. For stranded RNA-seq, cDNA libraries were prepared using an NEBNext^®^ Ultra™ Directional RNA Library Prep Kit (NEB, UK) according to the manufacturer’s instructions. Briefly, the mRNA was isolated and fragmented into 250–450 bp before synthesis of the first strand cDNA. The second strand of cDNA was then synthesized by adding dUTP as marker. Finally, the double strand cDNA was digested with Uracil—DNA Glycocasylase (UDG) before PCR reaction and sequencing. Thus, only the first strands of cDNA were kept and sequenced. The libraries were sequenced on a Hiseq 4000 (Illumina) using a paired-end run (2 × 150 bp).

The raw transcriptome data has been deposited at the NCBI database with Short Read Archive (SRA) accession numbers of SRR5279707- SRR5279711.

### Data processing and assembly

The raw reads were first quality-filtered by removing adapter sequences and reads with a quality under Q20 by using the FastQC tool (http://www.bioinformatics.bbsrc.ac.uk/projects/fastqc/). The total clean reads from the five libraries were then processed and *de novo* assembled with Trinity (Version 2.2.0) [59] using the default parameters. Trinity assembled the PE reads as contigs with a fixed *k*-mer value of 25. Contigs overlapped and reads that astride contigs were assigned into a same group, and then transcriptome was assembled by using *de Bruijn* graph strategy. The longest transcript extracted from each *de Bruijn* graph was defined as a Unigene, and these transcripts were used as reference sequences for subsequent analyses. The coding and protein sequences of unigenes were predicted by TransDecoder, a plug-in for Trinity (http://transdecoder.github.io/).

### Functional annotation of unigenes

The CDS and amino acid sequences of unigenes were aligned to the Gene Ontology (GO) database, the Cluster of Orthologous Groups of proteins (COG) database, the NCBI non-redundant (NR) protein database, the Swiss-Prot protein database, and the Kyoto Encyclopedia of Genes and Genomes (KEGG) database, respectively, by using BlastX program to identify functional genes (E-value < le^-5^).

### Identification and functional enrichment of DEGs

Gene expression levels were calculated and normalized using the FPKM method. DEGs between different libraries were identified using a Student’s *t*-test, and the *P*-value was adjusted using multiple testing procedures according to Benjamini and Yekutieli [60], by controlling the FDR. Finally, the significant DEGs were defined with the threshold of FDR < 0.005 and absolute value of log_2_ (fold-change) ≥ 1.

The GO and KEGG enrichments analyses for functional significance was performed using an ultra-geometric test with Benjamini-Hochberg correction [60]. GO terms for significant enrichment of DEGs were defined as corrected *P*-value < 0.05 when compared to the genome background. Pathways for significant enrichment of DEGs were defined as Q value < 0.05.

### Quantitative RT-PCR analysis

For quantitative RT-PCR (qRT-PCR) analysis, 1 μg of root total RNA from the control plants at 0 h 48 h, and 96 h and the Pb-treated *M*. *sativa* at 48 h and 96 h were treated with RNase-free DNase I before being used as a template for reverse transcription with a RevertAid First Strand cDNA Synthesis Kit (Thermo Scientific, USA). Quantitative RT-PCR analysis was performed using a SYBR^®^ Green master mixture (BioRad, USA) in a LightCycler^®^ 96 (Roche, USA). Genes and primers are listed in supplementary S1 Table.

## Supporting information

S1 Fig Sequencing saturation analysis (A) and distribution of unigene coverage (B) in each library.
(TIF)

S2 Fig A Venn diagram shows annotation of unigenes (A) and unigenes matching the 15 top species in the NR database.
(TIF)

S3 Fig COG functional classification of the assembled unigenes. (TIF)

S1 Table Primers used for qRT-PCR. (XLSX)

S2 Table The differentially expressed genes (DEGs) in Pb-treated roots of *M*. *sativa*. (xlsx)
(XLSX)

S3 Table GO enrichment of DEGs. (XLSX)

S4 Table KEGG enrichment of DEGs. (XLSX)

S5 Table Candidate genes involved in Pb stress response. (XLSX)
