# Supplementary figures and images for: Transcriptomic and physiological analyses of Medicago sativa L. roots in response to lead stress
Source: PLoS One. 2017 Apr 7;12(4):e0175307. doi: 10.1371/journal.pone.0175307 (PMC5384761; doi:10.1371/journal.pone.0175307)

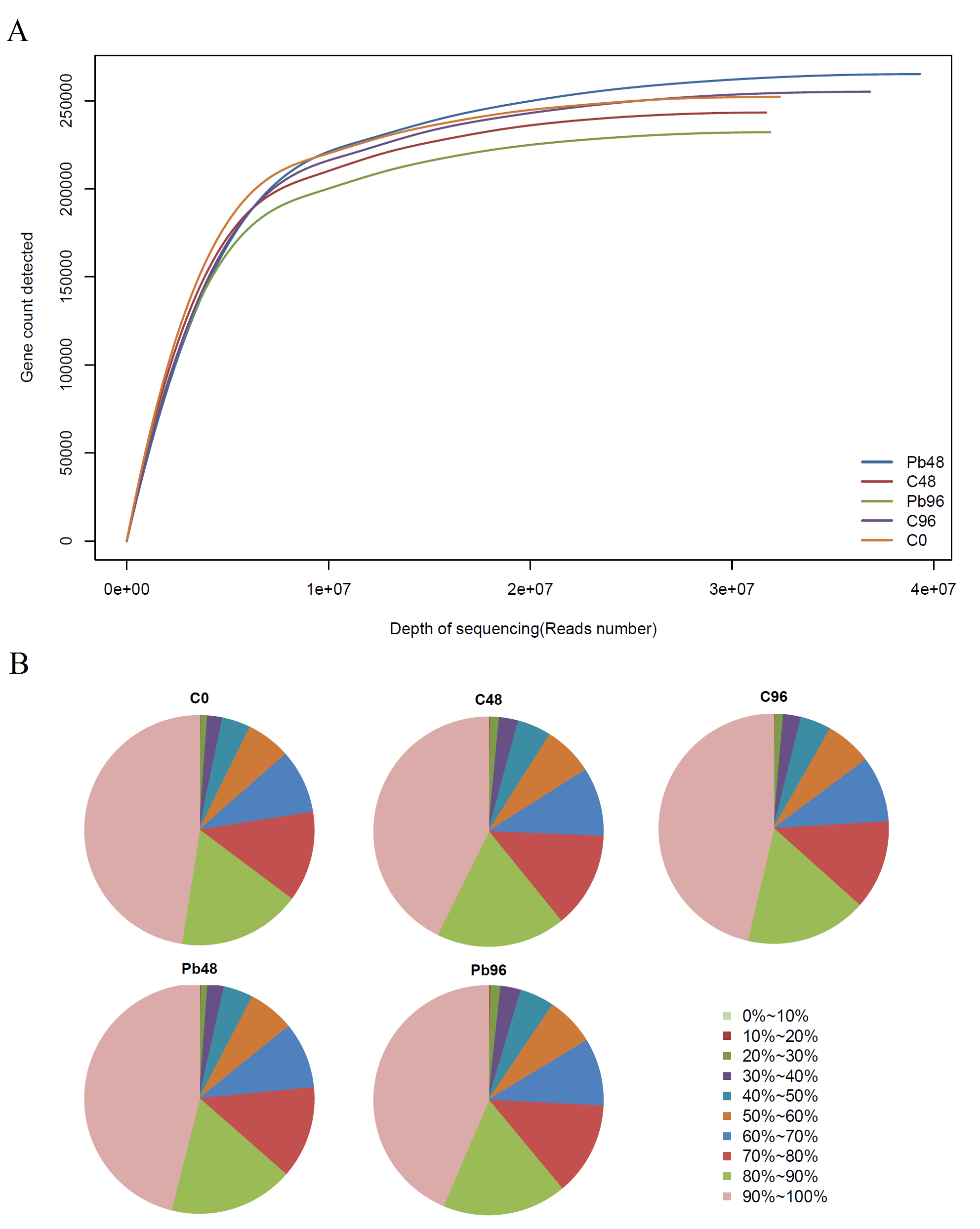

Supplement: S1 Fig — Sequencing saturation analysis (A) and distribution of unigene coverage (B) in each library. (TIF) [file pone.0175307.s001.tif]

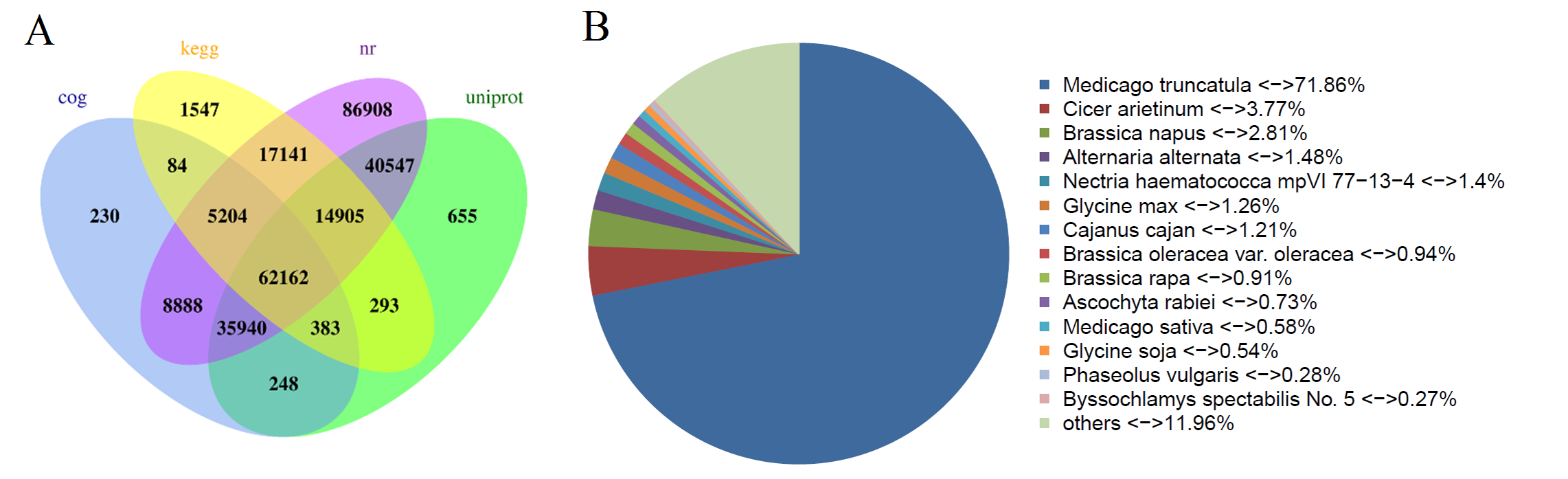

Supplement: S2 Fig — A Venn diagram shows annotation of unigenes (A) and unigenes matching the 15 top species in the NR database. (TIF) [file pone.0175307.s002.tif]

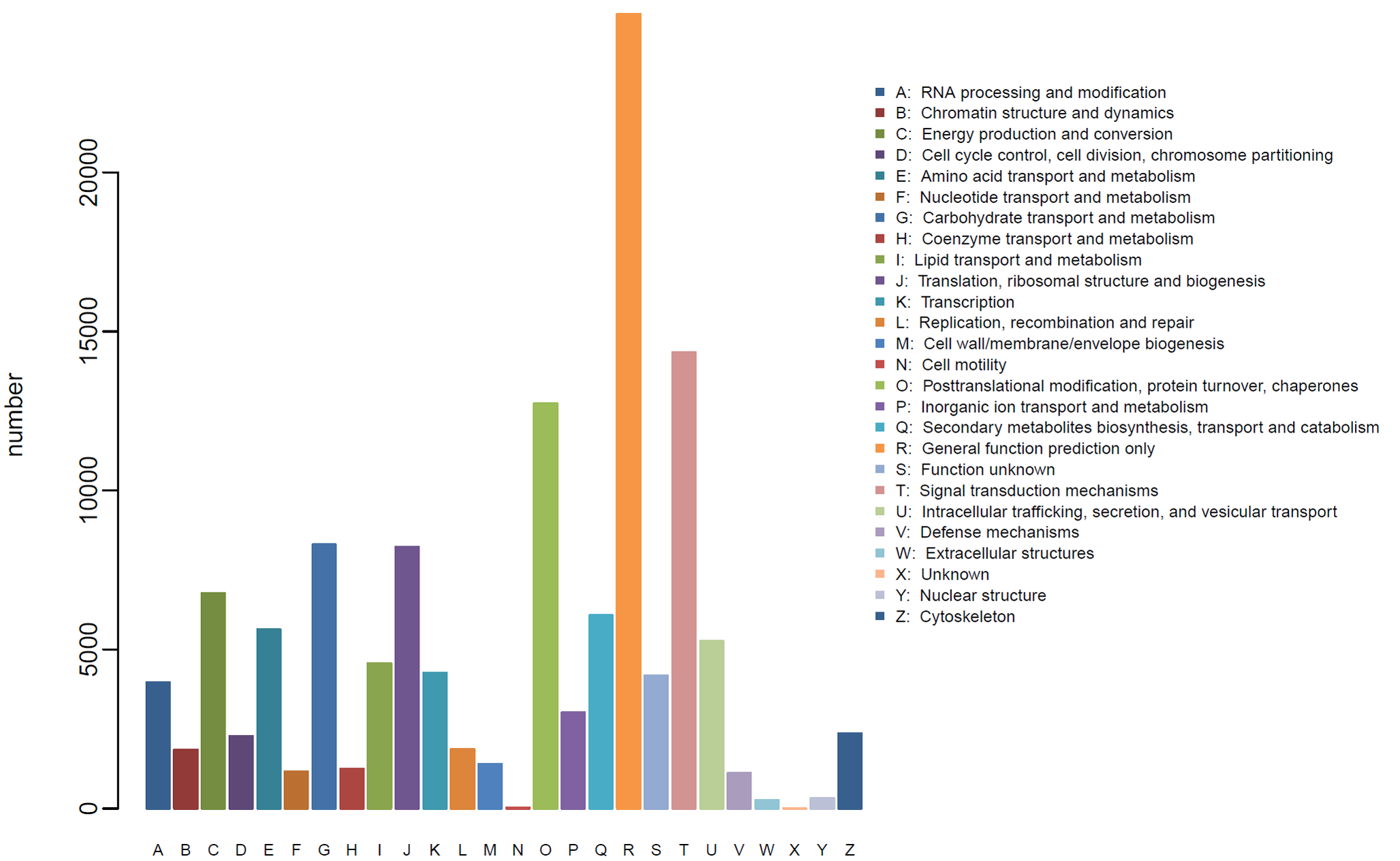

Supplement: S3 Fig — (TIF) [file pone.0175307.s003.tif]
